# Sorafenib with ASC‐J9^®^ synergistically suppresses the HCC progression *via* altering the pSTAT3‐CCL2/Bcl2 signals

**DOI:** 10.1002/ijc.30446

**Published:** 2016-11-09

**Authors:** Junjie Xu, Hui Lin, Gonghui Li, Yin Sun, Liang Shi, Wen‐Lung Ma, Jiang Chen, Xiujun Cai, Chawnshang Chang

**Affiliations:** ^1^ Chawnshang Chang Liver Cancer Center, Departments of General Surgery and Urology, Sir Run‐Run Shaw HospitalZhejiang UniversityHangzhouChina; ^2^ George Whipple Lab for Cancer Research, Departments of Pathology, Urology, Radiation Oncology and The Wilmot Cancer CenterUniversity of Rochester Medical CenterRochesterNYUSA; ^3^ Sex Hormone Research CenterChina Medical University/HospitalTaichungTaiwan

**Keywords:** Sorafenib, ASC‐J9^®^, hepatocellular carcinoma, synergism

## Abstract

Sorafenib is currently used as a standard treatment to suppress the progression of hepatocellular carcinoma (HCC), especially in advanced stages. However, patients who receive Sorafenib treatment eventually develop resistance without clear mechanisms. There is a great need for better efficacy of Sorafenib treatment in combination with other therapies. Here, we demonstrated that the treatment combining Sorafenib with ASC‐J9^®^ could synergistically suppress HCC progression *via* altering cell‐cycle regulation, apoptosis and invasion. Mechanism dissection suggests that while Sorafenib impacts little or even slightly increases the activated/phosphorylated STAT3 (p‐STAT3), a key stimulator to promote the HCC progression, adding ASC‐J9^®^ significantly suppresses the p‐STAT3 expression and its downstream genes including CCL2 and Bcl2. Interrupting these signals *via* constitutively active STAT3 partially reverses the synergistic suppression of Sorafenib‐ASC‐J9^®^ combination on HCC progression. *In vivo* studies further confirmed the synergistic effect of Sorafenib‐ASC‐J9^®^ combination. Together, these results suggest the newly developed Sorafenib‐ASC‐J9^®^ combination is a novel therapy to better suppress HCC progression.

## Abstract 2

**What's new?**

Sorafenib is currently a standard treatment to suppress the progression of hepatocellular carcinoma (HCC). STAT3 activation may however play a role in the development of Sorafenib resistance. Following earlier studies suggesting that ASC‐J9^®^ may alter activated p‐STAT3 signals to suppress prostate cancer metastasis, here the authors found that combining Sorafenib with ASC‐J9^®^ may synergistically suppress HCC progression. Sorafenib had little impact on p‐STAT3, whereas ASC‐J9^®^ significantly suppressed p‐STAT3 expression and its downstream genes, including CCL2 and Bcl2. Clinical studies using human HCC samples also demonstrated that higher expression of p‐STAT3 might be linked to the lower response to Sorafenib treatment.

**Abbreviations**
AR: androgen receptor
ATCC: American type culture collection
CI: combination index; FDA: U.S. Food and Drug Administration; HCC: hepatocellular carcinoma
IVIS: *in vivo* imaging system
PI: propidium iodide
RFS: recurrence‐free survival.

Hepatocellular carcinoma (HCC) incidence is increasing in the United States and elsewhere and is the sixth most prevalent neoplasm worldwide.^1^, ^2^ HCC is more effectively treated in the early stage upon early diagnosis. However, for advanced HCC, most therapies, including targeted chemotherapies, are still far from satisfactory.^3^

Sorafenib, the first drug approved for advanced HCC, is a tyrosine kinase inhibitor that targets VEGFR2 and Raf kinase.^4^ While Sorafenib treatment showed survival benefits in Phase III clinical studies,^5^, ^6^ many HCC patients still failed to respond, or developed resistance after being treated for several months.^7^

ASC‐J9^®^, the first AR (androgen receptor) degradation enhancer^8^, ^9^, ^10^ that could selectively degrade AR in selective cells, has been demonstrated to be able to suppress several AR‐related tumors including prostate, bladder, kidney and liver with few side effects.^11^, ^12^, ^13^ It has also been shown that ASC‐J9^®^ could have AR independent effects such as direct inhibition of STAT3 phosphorylation in AR‐negative PCa cells.^14^ Its potential influence over Sorafenib therapy, however, remains unclear.

STAT3, a cell proliferation‐related transcription factor that modulates many genes related to apoptosis, cell cycle and epithelial‐to‐mesenchymal transition (EMT),^15^, ^16^ was constitutively activated (through tyrosine phosphorylation) in many tumors including HCC.^17^, ^18^ Interestingly, recent studies indicated that p‐STAT3 (the phosphorylated and active form of STAT3) might be increased after chronic exposure to Sorafenib treatment in HCC cell lines, suggesting the deregulation of p‐STAT3 signals might be linked to Sorafenib resistance and/or relapse.^19^ Interestingly, our early studies of ASC‐J9^®^ in prostate cancer suggested that ASC‐J9^®^ could function through altering the p‐STAT3 signals to suppress prostate cancer metastasis,^14^ it remains unknown if such impact will alter the efficacy of Sorafenib therapy on HCC.

Here, we found a more effective therapy of combining sorafenib with ASC‐J9^®^ to synergistically suppress the HCC progression by altering pSTAT3‐CCl2/Bcl2 signals.

## Materials and Methods

### Materials

Sorafenib was purchased from Santa Cruz Biotechnology (Dallas, TX). ASC‐J9^®^ was purchased from AndroScience Corp (Solana Beach, CA).

### Tissue samples

For mouse studies, we collected all the livers of the mice and carefully examined the HCC nodules in them by H&E staining, and at least one nodule per liver was included although some of them were quite small for treatment groups. For clinical samples, tissue microarray (Super Biotek, Shanghai, China) was applied with a total of 80 HCC samples from the patients treated with Sorafenib. Among the samples, 3 of them lack the recurrence information and 2 of them had no cell nuclear staining. Thus, a total of 75 samples were included in this study. The demographic and clinical information of the patients were listed in **Supporting Information Table S1**.

### MTT cell viability assay and synergism evaluation

Cells were seeded in 24‐well plates (5 × 10^3^ cells/well) and incubated overnight for attachment, then they were treated with indicated doses of drugs in normal media for 48 hr. After treatment, the media were replaced with MTT (0.5 mg/ml) at 37°C for at least 1 hr. After removal of excess MTT, the cells were lysed with 500 µl dimethyl sulfoxide (DMSO) per well, and absorbance at 570 nm was measured and the values of 50% inhibition concentration (IC50) for each drug were determined by Compusyn software (ComboSyn, Inc.). The combination index (CI) value was determined from the fraction‐affected value of each combination according to the Chou–Talalay method using CompuSyn software (ComboSyn, Inc.) and a CI value below 1 represents synergism.^20^

### PI/annexin V apoptosis assay

HCC cells were plated in 6‐well plates at 2 × 10^5^ (HA22T) or 5 × 10^5^ (SKhep1) cells/well. Following the designated treatments for 24 (SKhep1) or 48 (HA22T) hr, all cells including both floating and trypsinized (0.25%Trypsin, without EDTA, from Gibco) attached cells were collected and washed with PBS. The apoptotic cells were detected by Annexin V Apoptosis Detection Kit FITC (eBioscience, San Diego) by staining with Annexin V‐FITC and PI according to the supplier's instructions. Viable and dead cells were detected by a BD LSRII flow cytometer (BD Biosciences). Blue B 515 and Green E 575 channels were applied for Annexin V‐FITC and PI, respectively.

### Lentiviral‐based gene delivery

By substituting cysteine residues for A661 and N663 of the Stat3 molecule that allowed for sulfhydryl bonds to form between Stat3 monomers and render the molecule capable of dimerizing without a phosphate on Y705,^21^ the constitutive activated STAT3 was cloned into pWPI backbone to get the pWPI‐Flag‐Stat3‐C plasmid and sequenced for confirmation purpose. (The mutant sequence is shown on **Supporting Information Table S2.**) The pWPI/pWPI‐Flag‐Stat3‐C, the psAX2 packaging plasmid, and pMD2G envelope plasmid, were then transfected into 293T cells using the standard calcium phosphate transfection method for 48 hr to get the lentivirus soup, which was collected and concentrated by density gradient centrifugation, then frozen in −80°C for later use in HA22T and SKhep1 cells infections.

### *In vivo* studies

Thirty‐two 6–8 weeks old athymic nude male mice were purchased from NCI. Intrahepatic injections of 1 × 10^6^ SKhep1‐luc cells/100 μl serum‐free DMEM and matrigel (1:1) were performed on each nude mouse. Cells were first prepared as stable luciferase clones by stable infection of Luciferase lentivirus and were selected with G418 and expanded in culture. One month later, the mice were divided into experimental groups according to tumor size following *in vivo* imaging (IVIS Spectrum, Caliper Life Sciences, Hopkinton, MA) after injecting 150 mg/kg Luciferin in tail vein, to start the treatment with a similar mean size in each group: (*i*) Control; (*ii*) Sorafenib treatment alone; (*iii*) ASC‐J9^®^ treatment alone; (*iv*) ASC‐J9^®^ + Sorafenib treatment. The mice were treated with/without Sorafenib (30 mg/kg/mice; every another day, I.P.) and with/without ASC‐J9^®^ (75 mg/kg/mice; every another day, I.P.) for another month. All control mice received an equal volume of carrier solution by I.P. Tumor development/response was then monitored by IVIS once a week. The mice were sacrificed 4 weeks after treatment and tumors and any metastases were removed for studies. All animal studies were performed under the supervision and guidelines of the University of Rochester Medical Center Animal Care and Use Committee.

### H&E and immunohistochemical (IHC) staining

Tissues were fixed in 10% (v/v) formaldehyde in PBS, embedded in paraffin, and cut into 5 μm sections and used for H&E staining and IHC staining with specific primary antibodies against phospho‐STAT3 (p‐STAT3, Tyr705; Cell Signaling), MCP‐1 (Cell Signaling), and Bcl‐2 (Santa Cruz). To enhance antigen exposure, the slides were treated with 1 × EDTA at 98°C for 10 min for antigen retrieval. The slides were incubated with endogenous peroxidase blocking solution, and then were incubated with the primary antibody at 4°C overnight. After rinsing with Tris‐buffered saline, the slides were incubated for 45 min with biotin‐conjugated secondary antibody, washed, and then incubated with enzyme conjugate horseradish peroxidase (HRP)‐streptavidin. Freshly prepared DAB (Zymed, South San Francisco, CA) was used as substrate to detect HRP. Finally, slides were counter‐stained with hematoxylin and mounted with aqueous mounting media. Positive cells were calculated as the number of immunopositive cells × 100% divided by total number of cells/field in 10 random fields at 400× magnification. The slides were reviewed and scored by an experienced pathologist without the knowledge of patient outcome. The expression of pSTAT3 was assessed semiquantitatively as follows: negative (−) <5%, 5–25% (+, weak positive), 25–50% (++, positive) and >50% (+++, strong positive).^22^ Negative and weakly positive expressions were defined as low expression, while positive and strong positive expressions were defined as high expression.

### Statistical analysis

Data are expressed as mean ± SEM from at least three independent experiments. Statistical analyses involved Student's *t test* with GraphPad Prism 5 (GraphPad Software, Inc., La Jolla, CA). For *in vivo* studies, measurements of tumor metastasis among the four groups were analyzed through one‐way ANOVA coupled with the Newman‐Keuls test. *p* <0.05 was considered statistically significant. More detailed methods information please see **Supporting Information**.

## Results

### Sorafenib with ASC‐J9^®^ synergistically suppressed HCC cell proliferation

**Figure 1:**
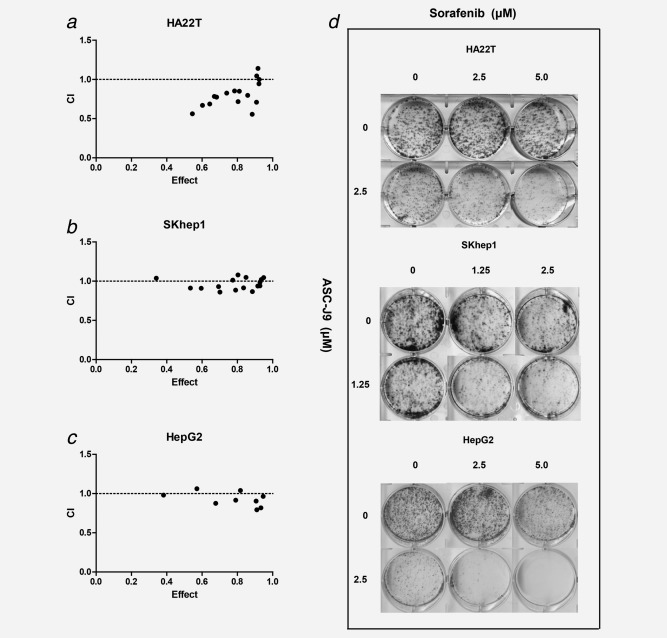
ASC‐J9^®^ combined with Sorafenib synergistically suppressed HCC cell viability. (*a*–*c*) Cells were seeded in 24‐well plates (5 × 10^3^ cells/well) and incubated overnight for attachment, and were then treated with indicated doses of ASC‐J9^®^ and Sorafenib in normal media for 48 hr. The impact of Sorafenib, ASC‐J9^®^ and the combination therapy on cell viability in HA22T, SKhep1 and HepG2 cells were determined by MTT assay. The CI values of each dose were calculated by the CompuSyn software and CI < 1 indicated synergism. (*d*) 6‐well dishes were seeded with 3 × 10^3^ viable cells and allowed to grow for 24 hr. The cells were then incubated in the presence or absence of Sorafenib or ASC‐J9^®^ and their combinations for 48 hr in complete media, washed with PBS gently, and allowed to grow in complete media for 10 days for colony formation. The colonies with >50 cells under microscope were counted. Three different independent experiments were performed.

Using MTT assay, we found Sorafenib or ASC‐J9^®^ alone suppressed HCC cell proliferation in a dose‐dependent manner in HA22T cells with IC50 value for ASC‐J9^®^ at 4.18 μM and Sorafenib at 7.32 μM. Importantly, the Sorafenib and ASC‐J9^®^ combination treatment had significantly enhanced efficacy to suppress HA22T cell proliferation (**Supporting Information Fig. S1A**). The CI values of each dose were calculated by the CompuSyn software and results suggested that at concentrations below the IC50 value, ASC‐J9^®^ exhibited a synergistic effect in combination with Sorafenib to suppress HA22T cells (Fig. 1 *a*; **Supporting Information Fig. S1D and Table S3**). The similar synergistic effect between Sorafenib and ASC‐J9^®^ was also observed in SKhep1 cells and HepG2 cells, indicating that this synergistic effect was not a cell line–specific effect (Figs. 1 *b* and 1 *c*; **Supporting Information Fig. S1B‐C, S1E‐F; Table S4 and S5**).

We further applied colony formation assay, a long term cell growth assay and obtained the similar results showing the combined Sorafenib and ASC‐J9^®^ treatment significantly enhanced the anti‐proliferative effects in HA22T, SKhep1 and HepG2 cells (Fig. 1 *d*).

Together, results from Figure 1 **, Supporting Information Fig. S1 and Table S3–S5** suggest that combined Sorafenib and ASC‐J9^®^ treatment synergistically suppressed cell viability with improved anti‐proliferative effects in HCC cells.

### ASC‐J9^®^ enhanced Sorafenib efficacy *via* inducing HCC cell‐cycle arrest and apoptosis

**Figure 2:**
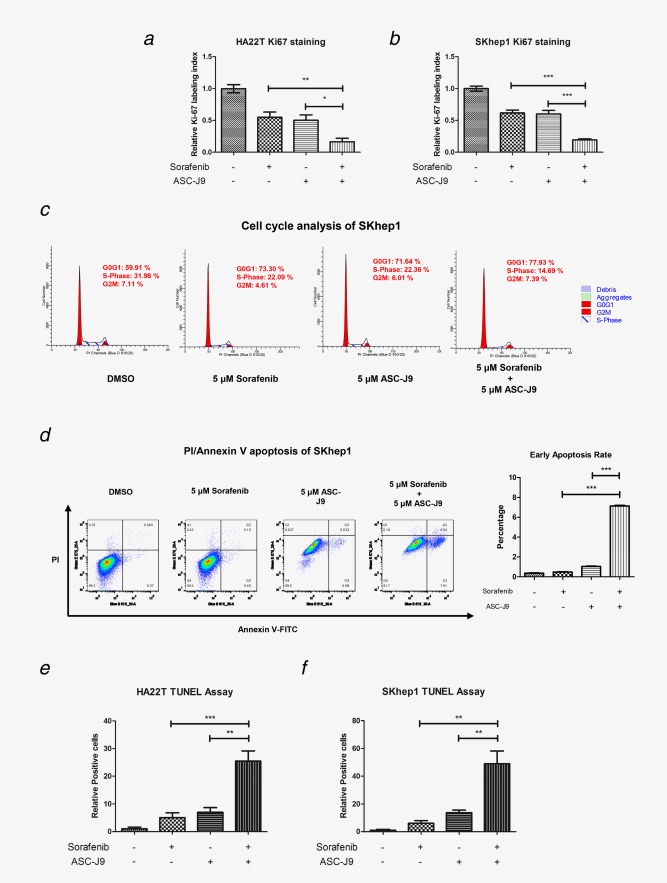
ASC‐J9^®^ enhanced Sorafenib efficacy to induce cell‐cycle arrest and apoptosis. (*a*, *b*) Ki‐67 immunofluorescence staining of HA22T and SKhep1 cells after designated treatments. Ki‐67–positive cells (the Ki‐67 labeling index) were calculated as the number of immunopositive cells × 100% divided by the total number of cells/field in 10 random fields at 100× magnification. (*c*) Cell cycle analysis of SKhep1 cells by PI/RNAse staining after designated treatment (5 × 10^4^ cells/experimental group). (*d*) PI/Annexin V apoptosis assay of SKhep1 cells by FACS after designated treatments (5 × 10^4^ cells/experimental group). Blue B 515 and Green E 575 channels were applied for Annexin V‐FITC and PI, respectively. Quantitation at right. (*e*, *f*) Apoptotic cell death was determined using TUNEL staining with an *in situ* Cell Death Detection Kit. TUNEL–positive cells were calculated as the number of positive cells × 100% divided by the total number of cells/field in 10 random fields at 100× magnification. *p < 0.05, **p < 0.01 and ***p < 0.005. [Color figure can be viewed at wileyonlinelibrary.com]

As Sorafenib and ASC‐J9^®^ were reported to induce cell‐cycle arrest and apoptosis in different cancer cells,^23^, ^24^ and our results suggested that this combination treatment might synergistically inhibit cell viability, we carried out cell‐cycle and apoptosis analysis to further characterize the Sorafenib and ASC‐J9^®^ combination effects on HCC cells. Ki67 staining assay showed that the combined therapy significantly suppressed the cell proliferation compared to Sorafenib or ASC‐J9^®^ alone in both HA22T and SKhep1 cells (Figs. 2 *a* and 2 *b* **, Supporting Information Figs. S2A and B**). Importantly, detailed cell cycle phase analysis through fluorescent activated cell sorting (FACS)^25^ also indicated that SKhep1 cell populations in the S and G2‐M phases were 14.69 and 7.39% in the Sorafenib and ASC‐J9^®^ combination group, while 22.09 and 4.61% in Sorafenib alone group, and 22.36 and 6.01% in ASC‐J9^®^ alone group (Fig. 2 *c*), suggesting that Sorafenib and ASC‐J9^®^ combination significantly induced G1 phase arrest compared with Sorafenib or ASC‐J9^®^ alone. Additionally, HA22T cells also showed significant cell cycle arrest (S phase arrest) when treated with Sorafenib and ASC‐J9^®^ combination compared with Sorafenib or ASC‐J9^®^ alone (**Supporting Information Fig. S3A**).

We also examined whether Sorafenib‐ASC‐J9^®^ combination enhanced the HCC cell apoptosis. PI/Annexin V apoptosis assay by FACS^25^ displayed little effect on cell apoptosis when treated with Sorafenib alone (0.45% of treated cells at early apoptosis rate compared with 0.37% in DMSO control group), and ASC‐J9^®^ alone induced apoptosis with 0.99% of treated cells at early apoptosis rate. In contrast, we found Sorafenib and ASC‐J9^®^ combination significantly induced apoptosis with 7.61% of treated cells at early apoptosis rate (Fig. 2 *d* **, Supporting Information Fig. S3B**). Due to drug color, ASC‐J9^®^ treatment itself would cause enhanced Blue B 515 and Green E 575 signals. Thus, we set the quadrant gate according to the main population in ASC‐J9^®^ group and Sorafenib‐ASC‐J9^®^ combination group. Apoptosis gauged by genomic DNA breaks through TUNEL assay^25^ further confirmed that Sorafenib‐ASC‐J9^®^ combination significantly induced apoptosis compared to Sorafenib or ASC‐J9^®^ alone in both HA22T and SKhep1 cells (Figs. 2 *e* and 2 *f* **, Supporting Information Fig. S4A and B**).

Together, results from Figure 2 **and Supporting Information Figs. S2–S4** suggest that the combined Sorafenib and ASC‐J9^®^ treatment can significantly induce cell‐cycle arrest and apoptosis while Sorafenib or ASC‐J9^®^ alone cannot.

### ASC‐J9^®^ enhanced Sorafenib efficacy to suppress HCC cell invasion

**Figure 3:**
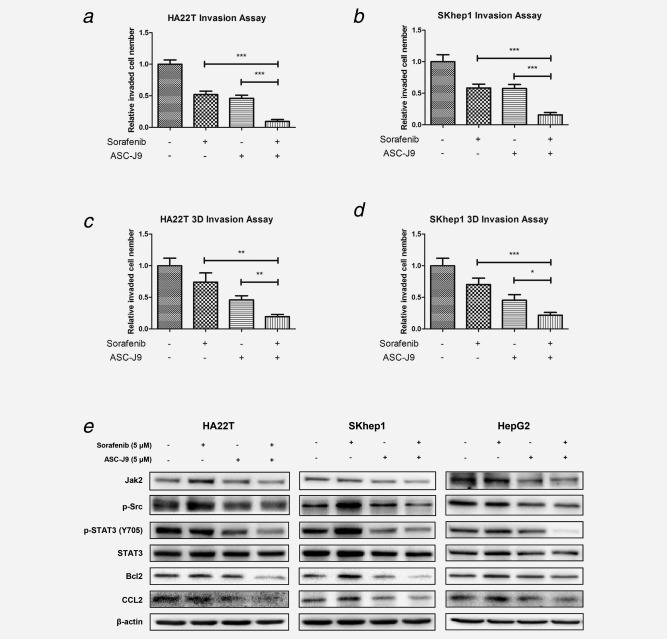
ASC‐J9^®^ enhanced Sorafenib efficacy to suppress HCC cell invasion. (*a*, *b*) Chamber‐transwell invasion assays were performed. 3 × 10^4^ HA22T cells or 5 × 10^4^ SKhep1 cells in serum free DMEM were plated into the upper chambers and 600 μl 10% FCS medium was placed in the lower chambers for incubation at 37°C in 5% (v/v) CO_2_ incubator for 24 hr. The invaded cells (to lower membrane surface) were counted in five randomly chosen microscopic fields (100×) of each experiment and pooled for statistical analysis. (*c*, *d*), 3D‐invasion assays were performed using HA22T and SKhep1 cells. The cells with protrusions were regarded as invaded cells and 10 random different fields under 200× magnification were counted for quantification. Each sample was run in triplicate and in multiple experiments. *p* < 0.05 was considered statistically significant. *p < 0.05, **p < 0.01 and ***p < 0.005. (*e*), ASC‐J9^®^ combined with Sorafenib synergistically suppressed HCC cell invasion and proliferation *via* altering p‐STAT3/CCLs and p‐STAT3/Bcl‐2 signals in three different HCC cell lines. HCC cells were plated in 6‐well plates at 2 × 10^5^ (HA22T) or 5 × 10^5^ (SKhep1 and HepG2) cells/well and treated as designated for 48 hr. After treatments, the cells were lysed and total protein was extracted for Western blot assay, which showed that Sorafenib and ASC‐J9^®^ combination therapy significantly suppressed p‐STAT3/CCL2 signals compared with ASC‐J9^®^ or Sorafenib alone in all three cell lines. Three different independent experiments were performed.

Previous studies demonstrated that ASC‐J9^®^ and Sorafenib could both suppress cancer cell invasion.^26^, ^27^ However, recent studies suggested that Sorafenib could also induce cellular invasion.^28^, ^29^ Using Boyden chamber assay,^30^ we found that the combined treatment suppressed HA22T cell invasion significantly in comparison to treatment with Sorafenib or ASC‐J9^®^ alone (Fig. 3 *a* **)**. Similar results were also obtained in HA22T cells (Fig. 3 *b*). Moreover, using the different 3D invasion assay, we also obtained similar results (Figs. 3 *c* and 3 *d*).

Together, results from Figure 3 suggest that combining ASC‐J9^®^ with Sorafenib can better suppress the HCC cell invasion.

### ASC‐J9^®^ with Sorafenib suppressed HCC cell invasion and proliferation *via* altering the p‐STAT3/CCL2/Bcl‐2 signals in HCC cell lines

To dissect the potential molecular mechanism(s) how ASC‐J9^®^ combined with Sorafenib suppressed HCC cell invasion and proliferation, we first focused on p‐STAT3 signals as early studies indicated that Sorafenib resistance in HCC might be linked to altered p‐STAT3 signals.^31^, ^32^ Interestingly, Lin *et al*. also found ASC‐J9^®^ could suppress prostate cancer cell invasion *via* inhibition of the p‐STAT3/CCL2 signals in an AR‐independent manner.^14^ Thus, the potential synergistic inhibition by these two compounds might derive from the enhanced inhibition of p‐STAT3 signaling, which is critical for tumor progression. We first examined the impact of Sorafenib and/or ASC‐J9^®^ on the expression of p‐STAT3 and STAT3 in HCC HA22T cells, and results revealed that Sorafenib alone led to little decrease of p‐STAT3 (Y705), while ASC‐J9^®^ suppressed p‐STAT3 (Y705) expression (Fig. 3 *e*). Importantly, the Sorafenib and ASC‐J9^®^ combination significantly suppressed p‐STAT3 (Y705) expression in HA22T cells (Fig. 3 *e*). We further examined their influence on pSTAT3 downstream target genes including CCL2 and Bcl2,^33^ and results showed that the combined Sorafenib and ASC‐J9^®^ treatment better suppressed the expression of both CCL2 and Bcl2 than Sorafenib or ASC‐J9^®^ alone in HA22T cells (Fig. 3 *e*).

Similar results were also obtained in SKhep1 and HepG2 cells showing combined Sorafenib and ASC‐J9^®^ treatment could significantly suppress the expression of p‐STAT3 (Y705) and its downstream targets including CCL2 and Bcl2 (Fig. 3 *e*). Interestingly, we found Sorafenib treatment increased the expression of p‐STAT3 (Y705) in SKhep1 and HepG2 cells (*vs*. little effect on HA22T cells), yet ASC‐J9^®^ alone still significantly suppressed the p‐STAT3 (Y705) expression, and combining ASC‐J9^®^ with Sorafenib also significantly suppressed the Sorafenib‐enhanced p‐STAT3 (Y705) expression. The Sorafenib‐enhanced downstream gene CCL2 and Bcl‐2 expressions were also reversed after addition of ASC‐J9^®^.

To further dissect the detailed mechanisms how Sorafenib and ASC‐J9^®^ impacted the p‐STAT3 level, we focused on several known upstream signals that might activate p‐STAT3, and results revealed that p‐Src and Jak2 were significantly increased after Sorafenib treatment, and the sorafenib‐ASC‐J9^®^ combination could reverse the increased expression of p‐Src and Jak2 in the 3 HCC cell lines (Fig. 3 *e*).

Together, results from Figure 3 *e* suggest that Sorafenib alone has either little effect or some increase on p‐Src/Jak2/p‐STAT3/CCL2/Bcl2 signals and addition of ASC‐J9^®^ enhances Sorafenib efficacy to suppress HCC cell invasion and proliferation *via* suppressing p‐Src/Jak2/p‐STAT3/CCL2/Bcl2 signals in the three different HCC cell lines.

### Constitutively active STAT3 mutant interrupted the synergistic effects of Sorafenib‐ASC‐J9^®^ suppression on HCC cell proliferation and invasion

**Figure 4:**
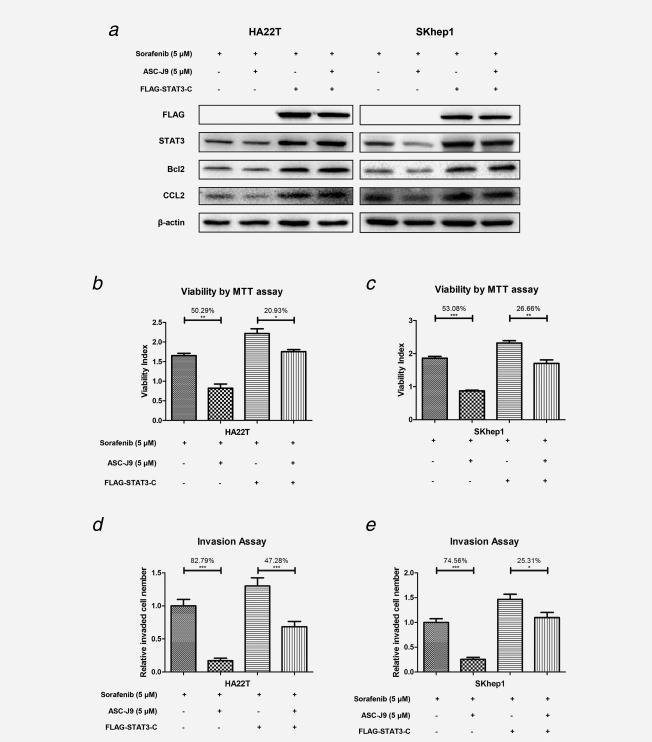
Constitutively activated mutant‐STAT3 interrupts the synergistic effects of Sorafenib and ASC‐J9^®^ suppression on HCC cell proliferation and invasion. (*a*) Western blot assay showed Sorafenib and ASC‐J9^®^ combination treatment significantly decreased the expression of STAT3 downstream gene CCL2 in vector HA22T and SKhep1 cells, yet this suppression was reversed significantly when we over‐expressed constitutively activated mutant‐p‐STAT3 (STAT3‐C). (*b*, *c*) The Sorafenib and ASC‐J9^®^ suppression of HA22T and SKhep1 cells proliferation reversed significantly when we overexpressed STAT3‐C. (*d*, *e*) The Sorafenib and ASC‐J9^®^ suppression of HA22T and SKhep1 cells invasion reversed significantly when we overexpressed STAT3‐C). *p* <0.05 was considered statistically significant. *p < 0.05, **p < 0.01 and ***p < 0.005.

To further prove the synergistic effect of the Sorafenib and ASC‐J9^®^ combination on suppression of HCC progression may require the inhibition of the p‐STAT3 (Y705) signals, we applied the interruption assay *via* expression of a constitutively active STAT3 (named as STAT3‐C), with Cys residues substituted by Ala‐661 and Asp‐663 within the STAT3, that allowed for sulfhydryl bonds to form between STAT3 monomers to constitutively activate STAT3.^21^ As shown in Figure 4 *a*, Sorafenib and ASC‐J9^®^ combined treatment significantly decreased the expression of p‐STAT3 downstream genes CCL2 and Bcl‐2 in HA22T and SKhep1 vector control cells (lane 1 *vs*. lane 2), yet this suppression was significantly reversed when we over‐expressed STAT3‐C (lane 3 *vs*. lane 4), suggesting that the suppression of Sorafenib and ASC‐J9^®^ combination on CCL2 and Bcl‐2 might need to function through phosphorylation inhibition of STAT3.

As expected, the suppression of Sorafenib and ASC‐J9^®^ combination on proliferation (Fig. 4 *b*) and cell invasion (Fig. 4 *c*; lane 1 *vs*. lane 2) was also significantly reversed upon over‐expressing STAT3‐C (lane 3 *vs*. lane 4) in HA22T cells with SKhep1 cells.

Together, results from Figure 4 suggest that the Sorafenib and ASC‐J9^®^ combination in HCC proliferation and invasion may need to function through the phosphorylation inhibition of STAT3.

### Sorafenib with ASC‐J9^®^ better suppressed HCC growth and metastasis in HCC *in vivo* mouse models

**Figure 5:**
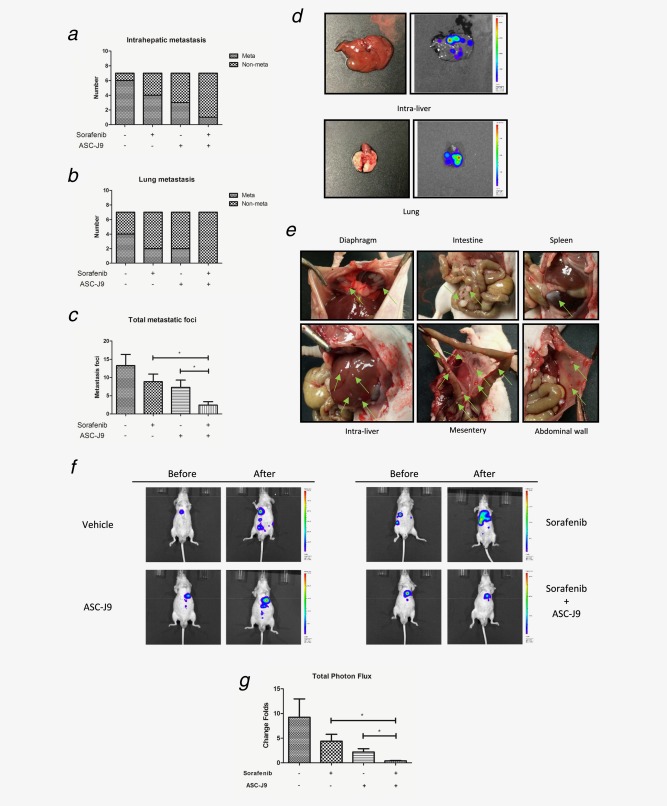
ASC‐J9^®^ combined with Sorafenib better suppressed HCC growth and metastasis *in vivo*. (*a*, *b*) IVIS imaging was used to determine the intrahepatic metastasis and lung metastasis. (*c*) Total metastatic foci were counted by autopsy. (*d*) Representative bioluminescent images of intrahepatic metastasis and lung metastasis. (*e*) Representative images of diaphragm, intestine, spleen, intra‐liver, mesentery and abdominal wall metastatic foci (green arrows). (*f*) Representative bioluminescent images before and after treatment in different treatment groups. (*g*) The change folds of total photon flux after treatment comparing to total photon flux before treatment were calculated using Living Image^®^ software (PerkinElmer).

**Figure 6:**
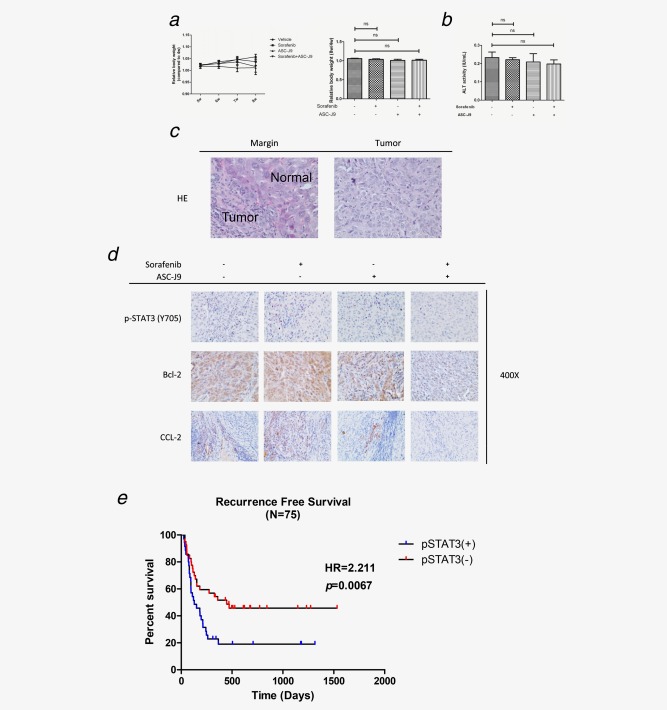
(*a*) Mouse body weights were measured once a week starting one month after xenografts. The relative body weight of each individual mouse was normalized to the body weight of 4 weeks. (*b*) The serum ALT activity (IU/ml) of each mouse before sacrificed was measured by ALT Colorimetric Activity Assay. (*c*) Representative HE staining images showing the xenografts tumor margin and tumor. (*d*) Representative images of IHC staining for p‐STAT3 (Y705), Bcl‐2 and CCL2 in different treatment groups. (*e*) Recurrence‐free survival curve of HCC patients who received Sorafenib treatment. The expression of pSTAT3 was assessed semiquantitatively as follows: negative (−) <5%, 5–25% (+, weak positive), 25–50% (++, positive) and >50% (+++, strong positive). Negative and weakly positive expression were defined as low expression, while positive and strong positive expression were defined as high expression. *p* < 0.05 was considered statistically significant. *p < 0.05, **p < 0.01 and ***p < 0.005.

To confirm the above *in vitro* cell lines data with the *in vivo* mouse model, we applied orthotopic HCC xenografts mouse model and found that Sorafenib or ASC‐J9^®^ alone could suppress HCC growth and metastasis. In addition, the Sorafenib and ASC‐J9^®^ combination resulted in much better efficacy to suppress HCC growth (tumor burden was evaluated by total photon flux, Fig. 5g) and metastasis (intrahepatic metastasis rate, lung metastasis rate and total metastasis foci, Figs. 5 *ae* **)**. Importantly, we found little body weight differences among these four groups **(**Fig. 6 *a* **)**, and the liver function determined by ALT activity showed little change after the administration of Sorafenib and/or ASC‐J9^®^ **(**Fig. 6 *b* **)**. IHC staining from these mice HCC tumors revealed similar patterns of change as those we found *in vitro* in p‐STAT3/CCL2/Bcl‐2 signals **(**Figs. 6 *c* and 6 *d* **)**.

### The expression of activated p‐STAT3 in HCC patients might link to the efficacy of Sorafenib therapy

Finally, we examined the p‐STAT3 expression by tissue microarray in 75 HCC patients before being treated with Sorafenib. It was found that p‐STAT3 (+) patients had worse recurrence‐free survival than p‐STAT3 (−) patients **(**Fig. 6 *e* **)**, suggesting that the expression of activated p‐STAT3 might play a key role to alter the efficacy of Sorafenib therapy.

## Discussion

HCC is the most common type of primary liver cancer and the leading cause of death among patients with cirrhosis.^34^ In the early stage, HCC is usually asymptomatic, which makes it difficult to diagnose and treat at the onset of the malignancy. Instead, most patients are diagnosed at intermediate or advanced stages, when therapies are less effective.^35^ The approval of Sorafenib by FDA as the first effective drug symbolized a great breakthrough on battling late stage HCC. The Sorafenib HCC Assessment Randomized Protocol trial in Europe^5^ and the Phase III Sorafenib Asia‐Pacific trial conducted in China, Taiwan and South Korea^6^ demonstrated that Sorafenib prolonged median overall survival time by 2.3–3 months and were well tolerated in patients with advanced HCC. However, the limited efficacy with negative side effects (such as anorexia, GI bleeds, hand‐foot syndrome, diarrhea, vomiting and squamous cell carcinoma,^5^, ^36^, ^37^ requires improvement of this therapy. Moreover, little responsiveness or development of Sorafenib resistance after treatment occurred in some HCC patients,^7^, ^38^ without a clear understanding of the mechanisms involved.

Early mechanism dissection suggested that Sorafenib might function through inhibiting Raf‐1 and B‐Raf and the receptor tyrosine kinase activity of VEGFRs 1, 2 and 3 and PDGFR‐β.^39^, ^40^ However, recent reports indicated that STAT3 activation also played a key role in the development of Sorafenib resistance in HCC cell lines.^19^, ^31^, ^32^ Our clinical studies using human HCC samples also demonstrated that higher expression of activated p‐STAT3 might indicate worse recurrence free survival under Sorafenib treatment. This finding is striking since we also found that adding 5 μM Sorafenib increased the expression of activated p‐STAT3 in 2 HCC cell lines, suggesting that increased p‐STAT3 expression might be an unwanted side effect for Sorafenib therapy to suppress HCC progression. We also noticed that Sorafenib was reported to be a potent inhibitor of STAT3 phosphorylation in some studies. In the Ref. 41, SK‐hep1 cells were treated with Sorafenib at 10 μM for 24 hr, and Ref. 42 reported the inhibition of p‐STAT3 in the condition of Sorafenib treatment (7.5 μM) for 24 hr. In our study, HA22T, SK‐hep1 and HepG2 cell lines were treated with Sorafenib (5 μM) for 48 hr, and we found p‐STAT3 slightly increased in HepG2 cells, significantly increased in SK‐hep1 cells and slightly decreased in HA22T cells, indicating that different conditions and cell origins might respond differently to Sorafenib treatment. We identified the particular biological condition (moderate dose of Sorafenib), which could give rise to the increase of p‐STAT3 and the following possible resistance to Sorafenib. Further mechanistic investigations might be necessary to fully understand the different behaviors of HCC cells under Sorafenib treatment.

The above dissected mechanisms suggest combining 2 chemotherapy drug(s) that could suppress p‐STAT3 might be able to overcome the Sorafenib‐induced p‐STAT3 and unwanted side effects. A common means to enhance the efficacy of chemotherapy for cancer is to combine with other therapy targeted or otherwise. If there is synergistic interaction between these therapies, a lower dose of the drugs can be used to minimize negative side effects, and more importantly, to enhance efficacy as well as to repress potential drug resistance.^20^ In the case of advanced HCC, it is known that Sorafenib can synergize with agents to suppress the STAT3 signaling to enhance its inhibition of upstream kinases including Raf and other receptor tyrosine kinases.^19^, ^32^ The exact mechanism for such synergy is not clear. It is possible that Sorafenib can somehow increase STAT3 phosphorylation as a feedback mechanism to reduce its efficacy,^43^ and an inhibition of STAT3 activity thus can boost Sorafenib efficacy. Several reports have lent credence to such a possibility. For example, it has been reported that other agents, including JAK inhibitor, the direct activator of STAT3, can also synergize with Sorafenib to inhibit cancer progression^44^ as well as molecules YC‐1^45^ and SC‐59^46^ to enhance SHP1 to enhance STAT3 dephosphorylation. As our recent studies found ASC‐J9^®^, a small molecule that could enhance AR degradation,^47^, ^48^, ^49^, ^50^, ^51^, ^52^ could also function through AR‐independent pathways to suppress prostate cancer metastasis *via* inhibiting p‐STAT3 expression (14), we therefore combined Sorafenib with ASC‐J9^®^ to examine their potential efficacy to suppress HCC. The results show that ASC‐J9^®^ can suppress the Sorafenib‐induced p‐STAT3 expression in an AR‐independent manner, and the combined Sorafenib and ASC‐J9^®^ treatment yields better suppression of HCC progression. This also indicated that combining Sorafenib and ASC‐J9^®^ treatment can synergistically suppress HCC progression *via* inducing cell cycle arrest in either G1 or S phase in HCC cells, which might be due to different genetic backgrounds of different HCC cells resulting in suppression of HCC cell proliferation.

In summary, we find a new combined therapy with Sorafenib and ASC‐J9^®^ that can significantly enhance Sorafenib efficacy to better suppress HCC progression. Future successful clinical trials may help us to slow‐down or overcome the Sorafenib‐induced unwanted side effects due to inducing p‐STAT3 expression in HCC.

## Supporting information

Supporting Information Figure 1.

Supporting Information Figure 2.

Supporting Information Figure 3.

Supporting Information Figure 4.

Supporting Information Tables.

Supporting Information
