# Supplementary material for: Sorafenib with ASC‐J9® synergistically suppresses the HCC progression via altering the pSTAT3‐CCL2/Bcl2 signals
Source: Int J Cancer. 2016 Nov 9;140(3):705–17. doi: 10.1002/ijc.30446 (PMC5215679; doi:10.1002/ijc.30446)
Supplement: Supplementary file 1 — Supporting Information Figure 1. [file IJC-140-705-s001.pptx]

## Slide 1
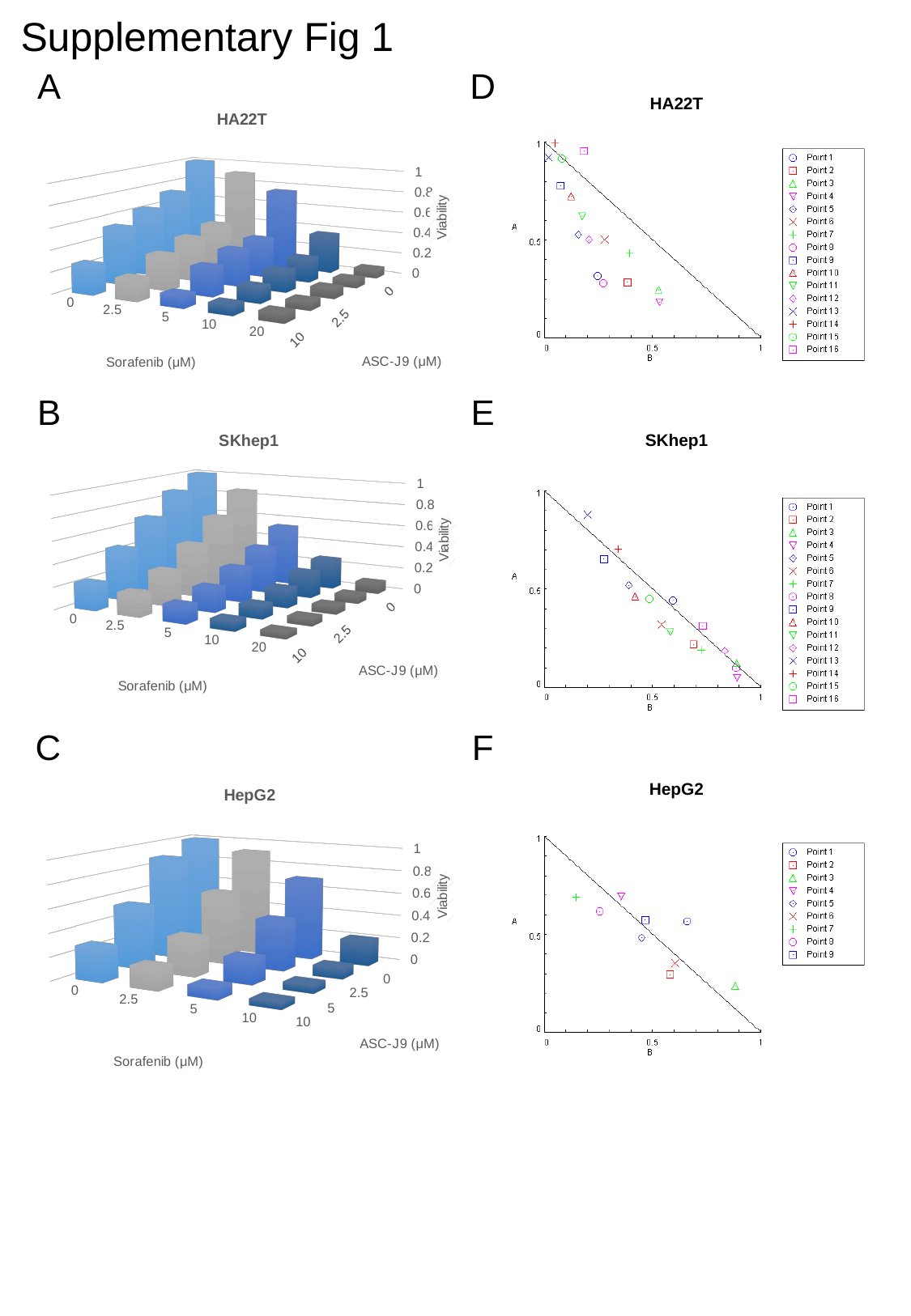

Supplementary Fig 1
A
D
HA22T
[unsupported chart]
B
E
SKhep1
[unsupported chart]
F
C
HepG2
[unsupported chart]
