# Supplementary material for: Sorafenib with ASC‐J9® synergistically suppresses the HCC progression via altering the pSTAT3‐CCL2/Bcl2 signals
Source: Int J Cancer. 2016 Nov 9;140(3):705–17. doi: 10.1002/ijc.30446 (PMC5215679; doi:10.1002/ijc.30446)
Supplement: Supplementary file 3 — Supporting Information Figure 3. [file IJC-140-705-s003.pptx]

## Slide 1
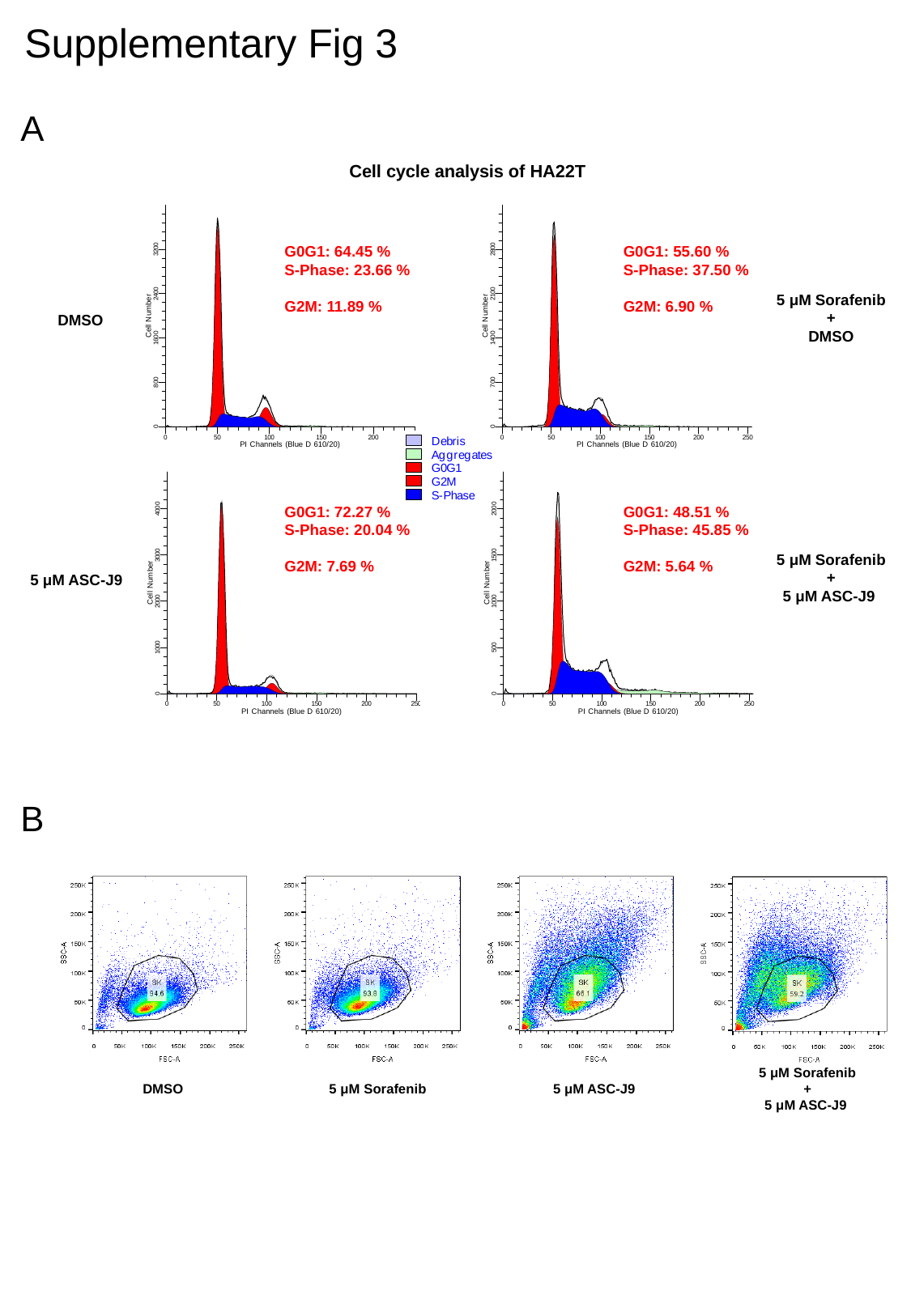

Supplementary Fig 3
A
Cell cycle analysis of HA22T
G0G1: 64.45 %
S-Phase: 23.66 %
G2M: 11.89 %
G0G1: 55.60 %
S-Phase: 37.50 %
G2M: 6.90 %
5 μM Sorafenib
 +
DMSO
DMSO
G0G1: 72.27 %
S-Phase: 20.04 %
G2M: 7.69 %
G0G1: 48.51 %
S-Phase: 45.85 %
G2M: 5.64 %
5 μM Sorafenib
 +
5 μM ASC-J9
5 μM ASC-J9
B
5 μM Sorafenib
 +
5 μM ASC-J9
DMSO
5 μM Sorafenib
5 μM ASC-J9
