# Supplementary material for: Sorafenib with ASC‐J9® synergistically suppresses the HCC progression via altering the pSTAT3‐CCL2/Bcl2 signals
Source: Int J Cancer. 2016 Nov 9;140(3):705–17. doi: 10.1002/ijc.30446 (PMC5215679; doi:10.1002/ijc.30446)
Supplement: Supplementary file 4 — Supporting Information Figure 4. [file IJC-140-705-s004.pptx]

## Slide 1
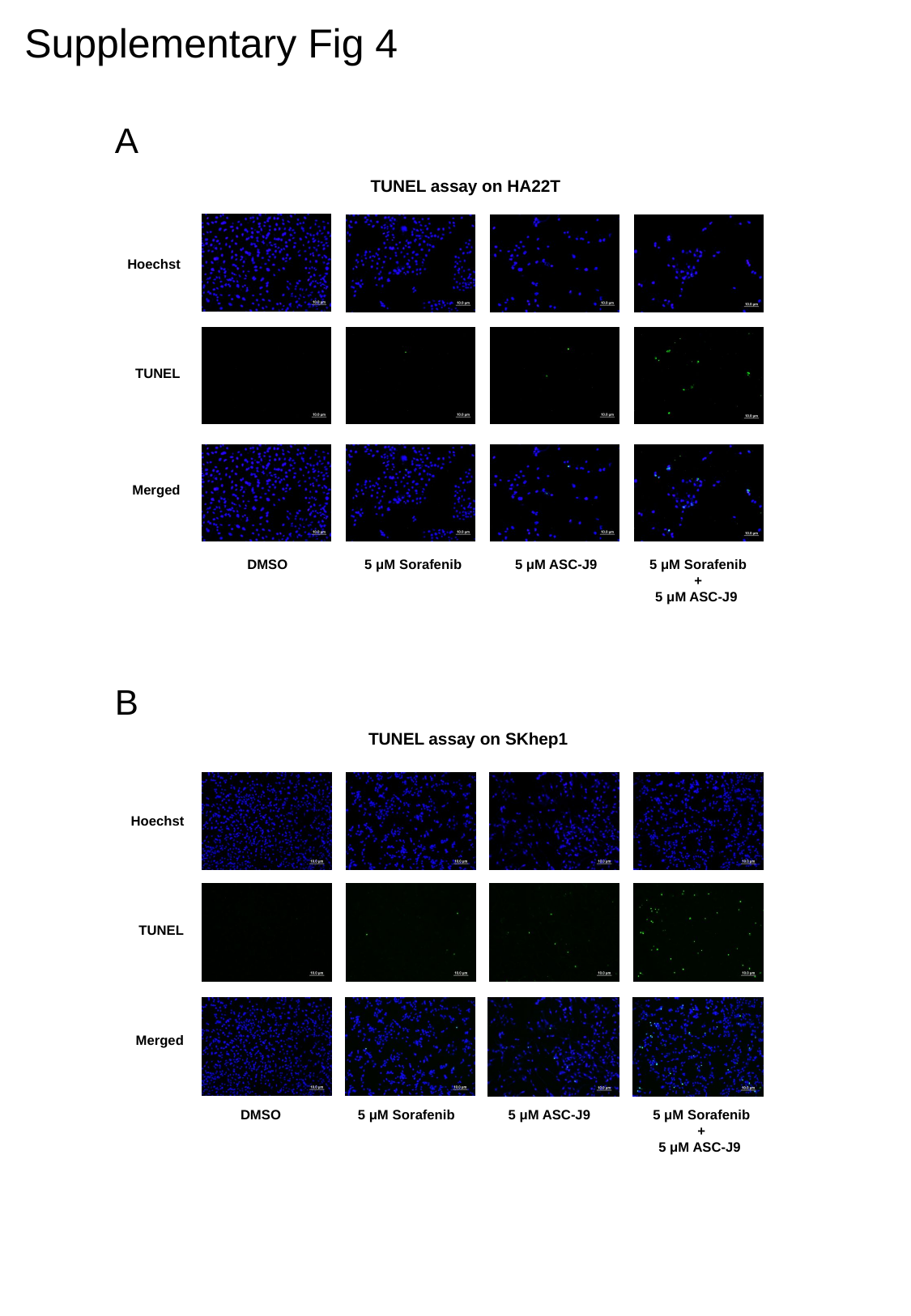

Supplementary Fig 4
A
TUNEL assay on HA22T
Hoechst
TUNEL
Merged
DMSO
5 μM Sorafenib
5 μM ASC-J9
5 μM Sorafenib
 +
5 μM ASC-J9
B
TUNEL assay on SKhep1
Hoechst
TUNEL
Merged
DMSO
5 μM Sorafenib
5 μM ASC-J9
5 μM Sorafenib
 +
5 μM ASC-J9
