# Supplementary material for: Sorafenib with ASC‐J9® synergistically suppresses the HCC progression via altering the pSTAT3‐CCL2/Bcl2 signals
Source: Int J Cancer. 2016 Nov 9;140(3):705–17. doi: 10.1002/ijc.30446 (PMC5215679; doi:10.1002/ijc.30446)
Supplement: Supplementary file 5 — Supporting Information Tables. [file IJC-140-705-s005.docx]

**Supplementary Table S1**

| Demographic and clinical information of the patients | | | | |
| --- | --- | --- | --- | --- |
|  | | **Numbers** | **Average pSTAT3 staining score** | **P value** |
| Total | | 75 | 1.573 | NA |
| Gender | Male | 71 | 1.549 | 0.3578 |
|  | Female | 4 | 2.000 |  |
| Age | <50 | 46 | 1.630 | 0.5144 |
|  | ≥50 | 29 | 1.483 |  |
| Multinodular | Yes | 5 | 0.6000 | 0.0163 |
|  | No | 70 | 1.643 |  |
| Tumor size (cm) | <5 | 27 | 1.593 | 0.8960 |
|  | ≥5 | 48 | 1.563 |  |
| Lymphatic or distant metastasis | Yes | 4 | 1.250 | 0.4865 |
|  | No | 71 | 1.592 |  |
| ALT (IU/L) | Normal* | 32 | 1.531 | 0.7423 |
|  | Abnormal* | 43 | 1.605 |  |
| AFP (μg/L) | Normal** | 13 | 1.538 | 0.8851 |
|  | Abnormal** | 62 | 1.581 |  |
| HBV | Yes | 62 | 1.500 | 0.1441 |
|  | No | 13 | 1.923 |  |
| Blood vessel tumor thrombus | Yes | 21 | 1.667 | 0.5979 |
|  | No | 54 | 1.537 |  |

All the information were collected before the surgery. The inclusion criteria is the HCC patients (Child-Pugh A-B) in advanced stages or with high risk of recurrence (Blood vessel tumor thrombus, Multinodular, positive surgical margins, postoperative residual tumor, Lymphatic or distant metastasis, high AFP after surgery, etc.) or in early stage but was enrolled in particular clinical trials.

*Normal: ALT≤50 IU/L; Abnormal: ALT>50IU/L

**Normal: AFP≤25μg/L; Abnormal: AFP>25μg/L

**Supplementary Table S2**

| **pWPI-Flag-STAT3-C mutation sequence** |
| --- |
| >pWPI-Flag-STAT3-C-STAT3-Seq-4_F09.ab1 NNNNNNNNNNNNGNNNCTTTATCNGTAAGGAGCGGGAGCGGGCCATCTTGAGCACTAAGCCTCCAGGCACCTTCCTGCTAAGATTCAGTGAAAGCAGCAAAGAAGGAGGCGTCACTTTCACTTGGGTGGAGAAGGACATCAGCGGTAAGACCCAGATCCAGTCCGTGGAACCATACACAAAGCAGCAGCTGAACAACATGTCATTTGCTGAAATCATCATGGGCTATAAGATCATGGATTGTACCTGTATACTGGTGTCTCCACTGGTCTATCTCTATCCTGACATTCCCAAGGAGGAGGCATTCGGAAAGTATTGTCGGCCAGAGAGCCAGGAGCATCCTGAAGCTGACCCAGGCGCTGCCCCATACCTGAAGACCAAGTTTATCTGTGTGACACCAACGACCTGCAGCAATACCATTGACCTGCCGATGTCCCCCCGCACTTTAGATTCATTGATGCAGTTTGGAAATAATGGTGAAGGTGCTGAACCCTCAGCAGGAGGGCAGTTTGAGTCCCTCACCTTTGACATGGAGTTGACCTCGGAGTGCGCTACCTCCCCCATGTGAGGGCAAACTACGGGCTGCAGGAATTCCGCCCCCCCCCCCCTAACGTTACTGGCCGAAGCCGCTTGGAATAAGGCCGGTGTGCGTTTGTCTATATGTTATTTTCCACCATATTGCCGTCTTTTGGCAATGTGAGGGCCCGGAAACCTGGCCCTGTCTTCTTGACGAGCATTCCTAGGGGTCTTTCCCCTCTCGCCAAAGGAATGCAAGGTCTGTTGAATGTCGTGAAGGAAGCAGTTCCTCTGGAAGCTTCTTGAAGACAAACAACGTCTGTAGCGACCCTTTGCAGGCAGCGGAACCCCCCACCTGGNGACAGGTGCCTCTGCGGCCAAAAGCCACGTGTATAANATACACCTGCAAAGGCGGCACNACCCCAGTGCCACGTTGTGAGTTGGATAGTTGTGNAAGAGTCAAATGGCTCTNCNTCAAGCGTATTCANCAAGGNCTGANNNTGCCCANAGTACCCCATTGTANGGNTCTGATCTGGNNNCGNGCNNATGCTTTACATNNNTTNNNCNAGTNAAAACGTCTNNNCCNCNNNNNGGGNCNNNNTTTCNNGAAAANNCNATNNNANNCNTGNNNNNNNNGGNNNNNNNNNNNNNNGGNNNNNCNNCNGNNNNNNNNNGNNNNNNANNNNNNNANNNNCNNNNNNNCNGNNNNGGNNN |

**Supplementary Table S3**

CI Data for Non-Constant Combo in HA22T cells

| **Combination Number** | **Dose SOR** | **Dose J9** | **Effect*** | **CI** |
| --- | --- | --- | --- | --- |
| 1 | 2.5 | 1.25 | 0.54506 | 0.56206 |
| 2 | 2.5 | 2.5 | 0.60229 | 0.66904 |
| 3 | 2.5 | 5.0 | 0.68240 | 0.77402 |
| 4 | 2.5 | 10.0 | 0.80401 | 0.71655 |
| 5 | 5.0 | 1.25 | 0.64378 | 0.68649 |
| 6 | 5.0 | 2.5 | 0.66953 | 0.78351 |
| 7 | 5.0 | 5.0 | 0.73963 | 0.82618 |
| 8 | 5.0 | 10.0 | 0.88412 | 0.55596 |
| 9 | 10.0 | 1.25 | 0.78398 | 0.85326 |
| 10 | 10.0 | 2.5 | 0.81116 | 0.84891 |
| 11 | 10.0 | 5.0 | 0.85837 | 0.79696 |
| 12 | 10.0 | 10.0 | 0.90844 | 0.70876 |
| 13 | 20.0 | 1.25 | 0.92275 | 0.94476 |
| 14 | 20.0 | 2.5 | 0.90987 | 1.04498 |
| 15 | 20.0 | 5.0 | 0.92418 | 0.99884 |
| 16 | 20.0 | 10.0 | 0.91702 | 1.14081 |

CI, Combination Index; SOR, Sorafenib; J9, ASC-J9^®^

*The effect represented the decreased percentage of viability.

**Supplementary Table S4**

CI Data for Non-Constant Combo in SKhep1 cells

| **Combination Number** | **Dose SOR** | **Dose J9** | **Effect*** | **CI** |
| --- | --- | --- | --- | --- |
| 1 | 1.25 | 2.5 | 0.34107 | 1.03871 |
| 2 | 1.25 | 5.0 | 0.59546 | 0.90908 |
| 3 | 1.25 | 10.0 | 0.77399 | 1.01300 |
| 4 | 1.25 | 20.0 | 0.92828 | 0.94063 |
| 5 | 2.5 | 2.5 | 0.53406 | 0.91161 |
| 6 | 2.5 | 5.0 | 0.70124 | 0.86051 |
| 7 | 2.5 | 10.0 | 0.83540 | 0.91407 |
| 8 | 2.5 | 20.0 | 0.92879 | 0.98672 |
| 9 | 5.0 | 2.5 | 0.69350 | 0.93022 |
| 10 | 5.0 | 5.0 | 0.79051 | 0.88562 |
| 11 | 5.0 | 10.0 | 0.88545 | 0.86699 |
| 12 | 5.0 | 20.0 | 0.93602 | 1.02029 |
| 13 | 10.0 | 2.5 | 0.80341 | 1.07974 |
| 14 | 10.0 | 5.0 | 0.84933 | 1.04841 |
| 15 | 10.0 | 10.0 | 0.91589 | 0.93867 |
| 16 | 10.0 | 20.0 | 0.94943 | 1.04592 |

CI, Combination Index; SOR, Sorafenib; J9, ASC-J9^®^

*The effect represented the decreased percentage of viability.

**Supplementary Table S5**

CI Data for Non-Constant Combo in HepG2 cells

| **Combination Number** | **Dose SOR** | **Dose J9** | **Effect*** | **CI** |
| --- | --- | --- | --- | --- |
| 1 | 2.5 | 2.5 | 0.38191 | 0.98116 |
| 2 | 2.5 | 5.0 | 0.67741 | 0.87621 |
| 3 | 2.5 | 10.0 | 0.81746 | 1.03876 |
| 4 | 5.0 | 2.5 | 0.57082 | 1.06142 |
| 5 | 5.0 | 5.0 | 0.79108 | 0.91528 |
| 6 | 5.0 | 10.0 | 0.90616 | 0.90440 |
| 7 | 10.0 | 2.5 | 0.91041 | 0.79346 |
| 8 | 10.0 | 5.0 | 0.93378 | 0.81766 |
| 9 | 10.0 | 10.0 | 0.94618 | 0.96446 |

CI, Combination Index; SOR, Sorafenib; J9, ASC-J9^®^

*The effect represented the decreased percentage of viability.
