# Supplementary material for: Sorafenib with ASC‐J9® synergistically suppresses the HCC progression via altering the pSTAT3‐CCL2/Bcl2 signals
Source: Int J Cancer. 2016 Nov 9;140(3):705–17. doi: 10.1002/ijc.30446 (PMC5215679; doi:10.1002/ijc.30446)
Supplement: Supplementary file 6 — Supporting Information [file IJC-140-705-s006.doc]

**Supplementary Methods**

**1 *In vitro* cell culture/maintenance:** The human HCC cell line HA22T/VGH was purchased from the Food Industry Research and Development Institute in Taiwan (BCRC number: 60168), SKhep1 and HepG2 were purchased from the American Type Culture Collection (ATCC, Manassas, VA, USA). All the cell lines were cultured in Dulbecco's Modified Eagle's Media (Invitrogen, Grand Island, NY, USA) supplemented with 10% FBS (v/v), penicillin (25 units/ml), streptomycin (25 g/ml), 1% L-glutamine, and 10% fetal bovine serum (FBS). Both cell lines were cultured in a 5% (v/v) CO_2_ humidified incubator at 37℃.

**2 Colony formation assay:** 6-well dishes were seeded with 3 × 10^3^ viable cells and allowed to grow for 24 hours. The cells were then incubated in the presence or absence of Sorafenib, ASC-J9® and their combinations for 48 hours in complete media, washed with PBS gently, and allowed to grow in complete media for another 10 days. The colonies obtained were then washed gently with PBS and fixed in 4% paraformaldehyde for 20 min at room temperature and then washed with PBS followed by staining with 0.1% (w/v) crystal violet. The colonies with >50 cells under microscope were counted. Three different independent experiments were performed.

**3 Cell cycle analysis:** HCC cells were plated in 6-well plates at 2 × 10^5^ (HA22T) or 5 × 10^5^ (SKhep1) cells/well. Following the designated treatments for 48 hours, cells were trypsinized with Trypsin/EDTA (0.25%) (Gibco by **Life Technologies,** Grand Island, NY, USA) and then washed with PBS and fixed in ice-cold 75% ethanol overnight at −20°C. Fixed cells were washed and dissolved in RNAse and permeabilized with 0.1% Triton X-100 and subsequently stained with propidium iodide (PI) incubated at 37°C for at least 30 min. The DNA content of the cells (5 × 10^4^ cells per experimental group) was determined using a BD LSRII flow cytometer (BD Biosciences, Franklin Lakes, NJ, USA).

**4 C****ell immunofluorescence staining:** Ki-67 detects proliferating cells in G1, S, G2, and mitosis, but not in the G0 resting phase. 10^5^ cells were seeded on 4-well chamber slides. After 48 hours, cells were fixed with 4% paraformaldehyde for 15 minutes. After 30 minutes blocking with 2% bovine serum albumin in PBS, Ki-67 antibody (Cell Signaling, Danvers, MA, USA) or anti-rabbit secondary antibody, conjugated with Avertin for fluorescence, were added to the slides overnight in 4°C. Cells were then mounted with mounting solution containing Hoechst 33258 (Sigma-Aldrich, St. Louis, MO, USA) and pictures were taken with fluorescence microscopy and Ki-67–positive cells (the Ki-67 labeling index) were calculated as the number of immunopositive cells × 100% divided by the total number of cells/field in 10 random fields at 100× magnification

**5 Fluorescent In Situ Detection of DNA Fragmentation (TUNEL):** Apoptotic cell death was determined using TUNEL staining with an In Situ Cell Death Detection Kit (Roche Molecular Biochemicals, Mannheim, Germany), following the manufacturer's protocol. Morphological changes in HCC cells undergoing apoptosis were then detected by counterstaining with Hoechst 33258 (Sigma-Aldrich). The slides were examined by fluorescence microscopy. TUNEL–positive cells were calculated as the number of positive cells × 100% divided by the total number of cells/field in 10 random fields at 100× magnification.

**6 Invasion assay:** The invasion capability of HCC cells was determined by the chamber-transwell invasion assay. The upper chambers of 8 µm-pore-size polycarbonate membrane filters (Corning, Inc., Corning, NY) were pre-coated with diluted growth factor-reduced matrigel (1:14 serum free DMEM) (BD Biosciences). Before invasion assays, HCC cells were plated in 6-well plates at 2 × 10^5^ (HA22T) or 5 × 10^5^ (SKhep1) cells/well and treated as designated for 48 hours. Then the cells were harvested by trypsinization and 3 × 10^4^ HA22T cells or 5 × 10^4^ SKhep1 cells in serum free DMEM were plated into the upper chambers and 600 μl 10% FBS media was placed in the lower chambers for incubation at 37°C in 5% (v/v) CO_2_ incubator for 24 hours. In order to determine whether the decrease of invaded cell number was attributed to cell death, we seeded the same amount of cell in triplicated wells of a 24-well plate and cultured in serum-free medium. After incubation, the cells in the upper chamber were removed and membranes scrapped and the cells invaded into the lower part of the membranes were stained with 0.1% (w/v) crystal violet. The invaded cells were counted in five randomly chosen microscopic fields (100×) in each experiment and averaged. When we collected the invasion results, we performed the MTT assay on the triplicated wells at the same time to normalize. As we found, in serum-free medium, the proliferation of the HCC cells in different treatment groups differed not so much.

**7 3D invasion assay**: Briefly, 5 x 10^4^ cells in 3 ml media containing 2.5% Matrigel and 30 ng EGF were plated into the collagen/Matrigel mixture coated plate. After treating for 48 hours, the media were replenished and every 3 days afterwards for 10 days. The cells with protrusion were regarded as invaded cells and 10 random different fields under 200× magnification were counted.

**8 Western Blot Analysis:** Cells were lysed in lysis buffer and proteins (30 µg) were separated on 10–12% SDS/PAGE gel and then transferred onto PVDF membranes (Millipore, Billerica, MA). After blocking membranes, they were incubated with appropriate dilutions of specific primary antibodies against Jak2 (Cell Signaling), p-Src (Cell Signaling), β-actin (Sigma-Aldrich), Phospho-STAT3 (Tyr705) (Cell Signaling), STAT3 (Santa Cruz), MCP-1 (Cell Signaling), Bcl-2 (Santa Cruz), and FLAG (M2) (Sigma-Aldrich). The blots were incubated with HRP-conjugated secondary antibodies (Invitrogen, USA) and visualized using the ECL system (Thermo Fisher Scientific, Rochester, NY).

**9 Alanine Transaminase (ALT) Colorimetric Activity Assay:**

Alanine Transaminase Colorimetric Activity Assay Kit (Cayman, Ann Arbor, Michigan) was used according to manufacturer’s protocol. Briefly, the mice blood samples were collected by cardiac puncture after overanesthetization and allowed to clot for 30 minutes at room temperature without using an anticoagulant. The samples were then centrifuged at 2,000 × g for 15 minutes at 4 ^o^C. We then used the serum layers for assays. Absorbance A_340_ was measured and ALT activity (IU/ml) was calculated according to manufacturer’s protocol.

**Legends to supplementary figures:**

**Supplementary Figure S1** (A-C) Cells were seeded in 24-well plates (5 × 10^3^ cells/well) and incubated overnight for attachment, and were then treated with indicated doses of ASC-J9® and Sorafenib in normal media for 48 hours. The impact of Sorafenib, ASC-J9® and the combination therapy on cell viability in HA22T, SKhep1, and HepG2 cells were determined by MTT assay. (D-F) The normalized isobolograms for combination therapy were calculated by the CompuSyn software and each point represented a combination dose (See **Supplementary Table S3-5**). The points under the diagonal line indicate synergism. S/SOR, Sorafenib; J/J9, ASC-J9®

**Supplementary Figure S2** (A, B) Images of Ki-67 immunofluorescence staining of HA22T and SKhep1 cells after designated treatments.

**Supplementary Figure S3** (A) Cell cycle analysis of HA22T cells by PI/RNAse staining after designated treatments (5 × 10^4^ cells/experimental group). (B) PI/Annexin V apoptosis assay of SKhep1 cells by FACS after designated treatments (5 × 10^4^ cells/experimental group). Blue B 515 and Green E 575 channels were applied for Annexin V-FITC and PI respectively. Gate 1 of the FACS assay was shown.

**Supplementary Figure S4** (A, B) Images of TUNEL staining of HA22T and SKhep1 cells after designated treatments.
